# Strengthening bonds via RyR2 inhibition helps immune suppression

**DOI:** 10.1172/JCI172986

**Published:** 2023-12-15

**Authors:** Erienne G. Norton, Nicole M. Chapman, Hongbo Chi

**Affiliations:** 1 Department of Immunology and; 2 St. Jude Graduate School of Biomedical Sciences, St. Jude Children’s Research Hospital, Memphis, Tennessee, USA.

## Abstract

Foxp3-expressing Tregs employ multiple suppressive mechanisms to curtail conventional T cell (Tconv) responses and establish tissue homeostasis. How Foxp3 coordinates Treg contact–dependent suppressive function is not fully resolved. In this issue of the *JCI*, Wang and colleagues revealed that Foxp3-mediated inhibition of ryanodine receptor 2 (RyR2) led to strong Treg-DC interactions and enhanced immunosuppression. RyR2 depletion in Tconvs phenocopied this effect and equipped Tconvs with Treg-like suppressive function in multiple inflammatory or autoimmune contexts. This study provides molecular and therapeutic insights underlying how cell-cell contact limits immune reactivity.

## Treg suppressive mechanisms limit immune reactivity

**Figure 1:**
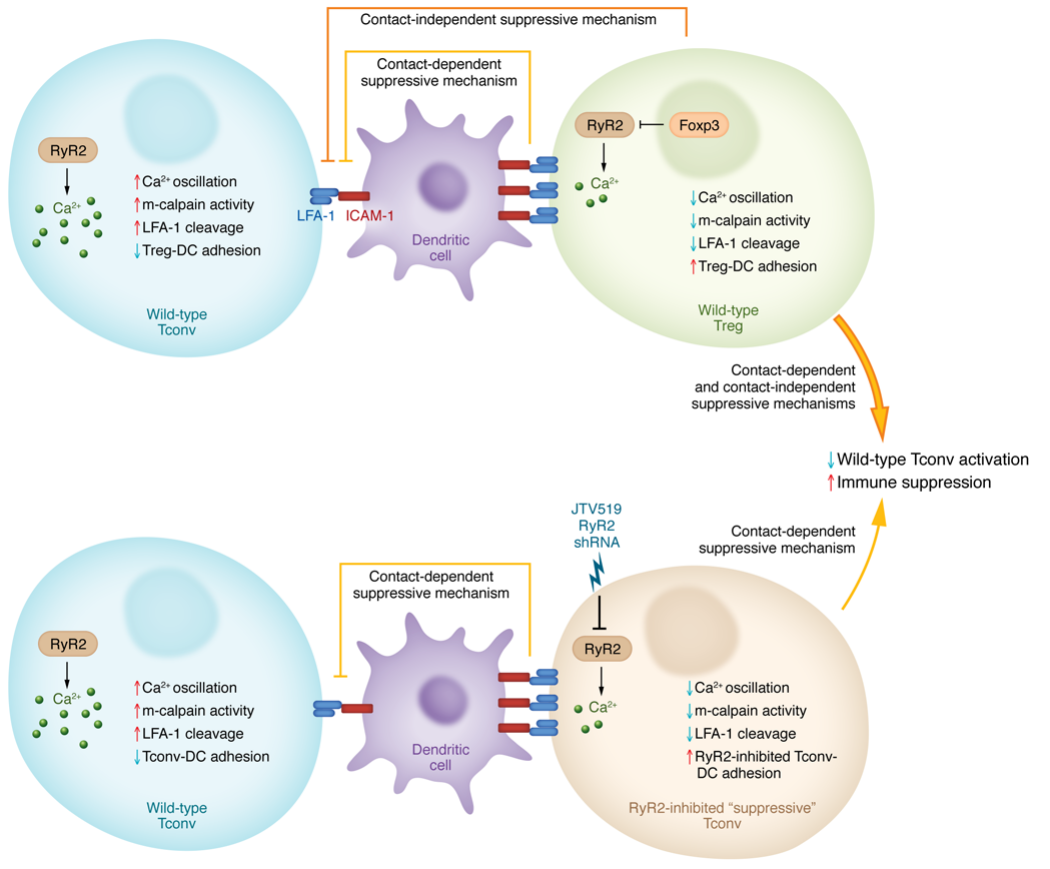
RyR2 controls the binding dynamics of Tregs and Tconvs with DCs and its inhibition facilitates immune suppression. Expression of the sarcoendoplasmic reticulum-localized calcium (Ca^2+^) release channel component RyR2 is high in conventional CD4^+^ T cells (Tconvs). RyR2 contributes to high basal Ca^2+^ oscillations and relatively weak adhesion to DCs, owing to increased m-calpain activation that promotes LFA-1 cleavage and disrupted LFA-1–ICAM-1 interactions. In Tregs, Foxp3 suppresses RyR2 expression, resulting in low basal Ca^2+^ oscillations and downstream m-calpain activity. Consequently, less LFA-1 is cleaved from the Treg surface, and Tregs strongly adhere to DCs via enhanced LFA-1–ICAM-1 interactions. Pharmacological inhibition using JTV519 or shRNA-mediated targeting of RyR2 in Tconvs phenocopies the contact-dependent immunosuppressive effects of Tregs without causing them to adopt a Treg-like program (e.g., Foxp3 expression). Ultimately, low RyR2 levels increase the interactions between Tregs or Tconvs and DCs, thereby promoting immune suppression. Of note, Tregs display higher suppressive function than RyR2-deficient Tconvs, likely due to their capacity to engage both cell contact–dependent and cell contact–independent mechanisms.

Tregs are a specialized subset of CD4^+^ T cells characterized by the expression of the master transcription factor, Foxp3, which orchestrates Treg development and function. Rather than inducing cytolytic or proinflammatory immune cell function, Tregs suppress immune responses under steady state and upon inflammatory challenges, thereby preventing tissue damage and other pathologies. To mediate these effects, Tregs utilize multiple suppressive mechanisms, including deprivation of IL-2 (1–3), secretion of inhibitory cytokines IL-10 and TGF-β (1, 3), and production of cytolytic granzymes (1, 3). Cell-cell contact also plays a crucial role in Treg-mediated suppression. For instance, Tregs express high levels of several coinhibitory receptors (e.g., TIGIT, PD-1, CTLA-4), which trigger suppressive signaling or removal of key stimulatory molecules expressed on DCs to limit conventional T cell (Tconv) activation (1, 3–5). Although Tregs strongly adhere to DCs via interactions between LFA-1 and ICAM-1 (6–8), it remains poorly understood whether and how Foxp3 orchestrates Treg contact–dependent suppressive function. Exploring the molecular basis may uncover therapies for tuning Tconv responses in autoimmune or allergic disease, organ or tissue transplantation, and cancer. In this issue of the *JCI*, Wang and authors report a Foxp3-regulated mechanism that facilitates strong Treg-DC interaction, which is associated with potent immunosuppression in murine models of autoimmunity, inflammation, and cancer (9) (Figure 1).

## RyR2 dictates Treg-DC contact-mediated suppressive function

Tregs have reduced activity of cysteine proteases called calpains, with the Treg-specific reduction in m-calpain activity mediating long-lived Treg-DC adhesion that is dependent on LFA-1 and ICAM-1 interactions (10). The protease activity of m-calpain is regulated by calcium in a poorly defined process. To explore the underlying mechanisms, Wang and colleagues first examined whether basal calcium oscillations were different in CD4^+^CD25^+^ Tregs versus CD4^+^CD25^–^ Tconvs and found a marked reduction in Tregs (9). Next, the authors compared the expression of multiple proteins involved in calcium regulation in Tregs versus Tconvs and revealed reduced expression of RyR2, a component of a Ca^2+^ release channel located within the sarcoendoplasmic reticulum, in Tregs. These results suggested that RyR2 expression was reciprocally associated with calcium oscillations and the strength of interactions with DCs in Tregs versus Tconvs. To test whether the reduction of RyR2 activity enhanced interactions between Tconvs and DCs, the authors next employed pharmacological inhibition (using JTV519, which prevents Ca^2+^ release) or shRNA-mediated depletion of RyR2 in Tconvs. These treatments reduced basal calcium oscillations and increased Tconv-DC interactions, with such observations being comparable with those in Tregs.

From a functional perspective, the authors showed that both Tregs, which possessed a low baseline expression of RyR2, and RyR2-deficient Tconvs were able to outcompete antigen-specific Tconvs for binding to antigen-bearing DCs in vitro. Such an effect was associated with reduced DC-mediated Tconv proliferation, indicative of an improved suppressive capacity in the absence of RyR2. To extend these observations to physiologically relevant settings in vivo, Wang and colleagues next employed adoptive transfer of RyR2-deficient Tconvs (versus wild-type Tconvs or wild-type Tregs) into multiple immune-mediated disease models, including infection or inflammatory models (herpes simplex virus-1 infection, ovalbumin-induced [OVA-induced] airway inflammation, and dextran sulfate sodium–induced colitis), a cancer model (MC38 adenocarcinoma), and an autoimmune model (scurfy mice). In all of these systems, RyR2 deficiency in Tconvs conferred protection against inflammation and immune hyperreactivity to a largely similar extent as Tregs (9). Collectively, the authors establish that RyR2-coordinated calcium oscillations dictate the capacity for Tregs and Tconvs to bind DCs, with lower RyR2 expression being associated with enhanced binding and improved suppressive function (Figure 1).

## Mechanistic regulation of RyR2 expression and functional effects

As Foxp3 expression is unique to Tregs, Wang and colleagues next examined whether Foxp3 suppressed RyR2 expression. They found that overexpression of Foxp3 in Tconvs and multiple cell lines reduced RyR2 transcription in vitro. Chromatin immunoprecipitation assays validated the binding of overexpressed and endogenous Foxp3 to the *Ryr2* locus. Using a luciferase reporter assay, the authors identified a Foxp3-specific binding site located approximately 200 base pairs upstream of the *Ryr2* start codon (9), indicating that Foxp3 directly bound to RyR2 to inhibit its expression.

To identify the mechanism by which RyR2 deletion in Tconvs promoted their suppressive function, the authors performed transcriptomic, epigenetic, and TCR repertoire analyses in RyR2-deficient Tconvs compared with wild-type Tconvs or Tregs. They found that RyR2-deficient Tconvs were more similar to wild-type Tconvs than Tregs (9), suggesting that RyR2 deficiency is not sufficient to reprogram Tconvs to adopt a bona fide Treg program. Additionally, T cell–specific deletion of RyR2 did not lead to any cell-intrinsic alterations in the thymic development or peripheral homeostasis of CD4^+^ T cells. Furthermore, there were no impairments on Treg homeostasis or changes in the expression of Treg-associated suppressive molecules, including CTLA-4, PD-1, TGF-β, or IL-10. Finally, Wang and colleagues also showed that RyR2-deficient Tconvs formed contacts with DCs independently of a specific antigen, suggesting an antigen-independent suppressive mechanism mediated by Treg-DC contacts that is distinct from the roles of TCR signals in promoting Treg functional fitness in vivo (11, 12). Thus, Foxp3-dependent repression of RyR2 is insufficient to orchestrate the full Treg suppressive program.

## Clinical implications of targeting RyR2 for immunotherapy

The therapeutic effects that RyR2-deficient Tconvs conveyed in multiple inflammatory and autoimmune models suggest that targeting of RyR2 activity may have therapeutic potential for autoimmune diseases, which are often associated with low numbers or dysfunction of Tregs (3, 13, 14). Indeed, boosting Treg numbers or suppressive function is an emerging strategy for disease therapy. For example, overexpression of Foxp3 in Tconvs is used to treat autoimmune conditions, such as immune dysregulation polyendocrinopathy enteropathy X-linked syndrome, as well as to maintain graft tolerance and prevent graft-versus-host disease (14). However, an unstable Treg-like intermediate cell population with effector activity can arise under such conditions, posing a risk for pathogenicity rather than the establishment of immune tolerance (14). Remarkably, Wang and authors found that inhibition of RyR2 induced a suppressive phenotype in Tconvs even without enforced Foxp3 expression. These observations, combined with the fact that RyR2 inhibition largely did not alter Tconv activation status in vivo and had minimal effect on their transcriptomic or epigenomic features, suggests that targeting RyR2 may promote Treg-like suppressive activity in Tconvs without compromising the functional fitness of conventional CD4^+^ T cells. Engineering antigen-specific Tregs, such as via synthetic manipulation of the TCR or the generation of cells expressing a chimeric antigen receptor, is also of great therapeutic interest due to their increased suppressive function and capacity for homing to inflammatory sites (13, 14). However, antigen-specific Tregs are present at low frequency and have poor proliferative potential, which creates hurdles to harness their therapeutic potential (13, 14). Thus, maximizing the efficacy of antigen-independent Treg-based immunotherapies is clinically relevant, making inhibition of RyR2 an intriguing candidate. It is noteworthy that RyR2 inhibitors, such as flecainide and dantrolene, are already used for the treatment of cardiac arrhythmias, so they may be repurposed to boost immunosuppression in autoimmunity; however, the possibility of affecting cells other than Tregs must be considered. Although the authors did not report on the impact of RyR2 overexpression in a tumor model, increasing RyR2 activity may decrease Treg suppressive function and enhance DC-mediated activation of Tconvs in the tumor microenvironment, where Tregs frequently impede antitumor immunity (15). Thus, targeting RyR2 has multiple potential immunotherapeutic applications.

## Conclusions and future directions

Wang and authors identified a link between Foxp3 and regulation of Treg-DC contact mediated by RyR2-regulated calcium oscillations, thereby providing important insights as to how calcium flux is regulated in Tregs to control their development and function (16, 17). Moreover, by showing that RyR2 regulates m-calpain activity, this study establishes spatially regulated calcium signals at the plasma membrane–endoplasmic reticulum junction in dictating cell-cell contact and adaptive immunity (9). The observation that RyR2 inhibition equips Tconvs with suppressive function (albeit with more limited capacity than Tregs in vivo) opens new opportunities for therapeutic targeting of the RyR2 pathway to heighten Tconv suppressive function in autoimmune diseases and graft-versus-host disease.

Interestingly, while RyR2-deficient Tconvs were functionally suppressive, mice that received wild-type Tregs had improved disease control and lower tissue inflammation scores than mice receiving RyR2-deficient Tconvs in both the scurfy and OVA-induced airway inflammation models (9). Therefore, despite the contact-based suppression that occurs upon inhibition of RyR2, the more abundant expression of suppressive molecules on wild-type Tregs than RyR2-deficient Tconvs suggests that RyR2-independent mechanisms are crucial for Treg suppressive capacity in vivo. Future studies are warranted to determine whether RyR2-coordinated Treg-DC contact cooperates with or facilitates other modes of Treg-mediated suppressive activity. For example, RyR2-regulated Treg contact with DCs may contribute to CTLA-4–mediated trans-endocytosis or trogocytosis of CD80 and CD86 or facilitate Treg-DC-Tconv interactions that limit and enhance IL-2–induced STAT5 activation in Tconvs and Tregs, respectively (2, 4, 5). It will also be interesting to explore whether RyR2 regulates spatiotemporal Treg interactions with other cell types, including B cells, tumor-associated myeloid populations, and stem cells, to promote Treg function in different inflammatory contexts or microenvironments (18–20). Finally, it remains important to assess whether gain of function for RyR2 in Tregs contributes to aberrant inflammation or autoimmunity or could be used therapeutically for the treatment of cancer (15). Overall, the findings from Wang and colleagues provide important insights into how Foxp3 orchestrates calcium-regulated cell-cell communication to facilitate Treg suppressive function in inflammatory diseases, cancer, and autoimmunity, which will be of strong clinical interest.

## Version 1 12/15/2023

Electronic publication
